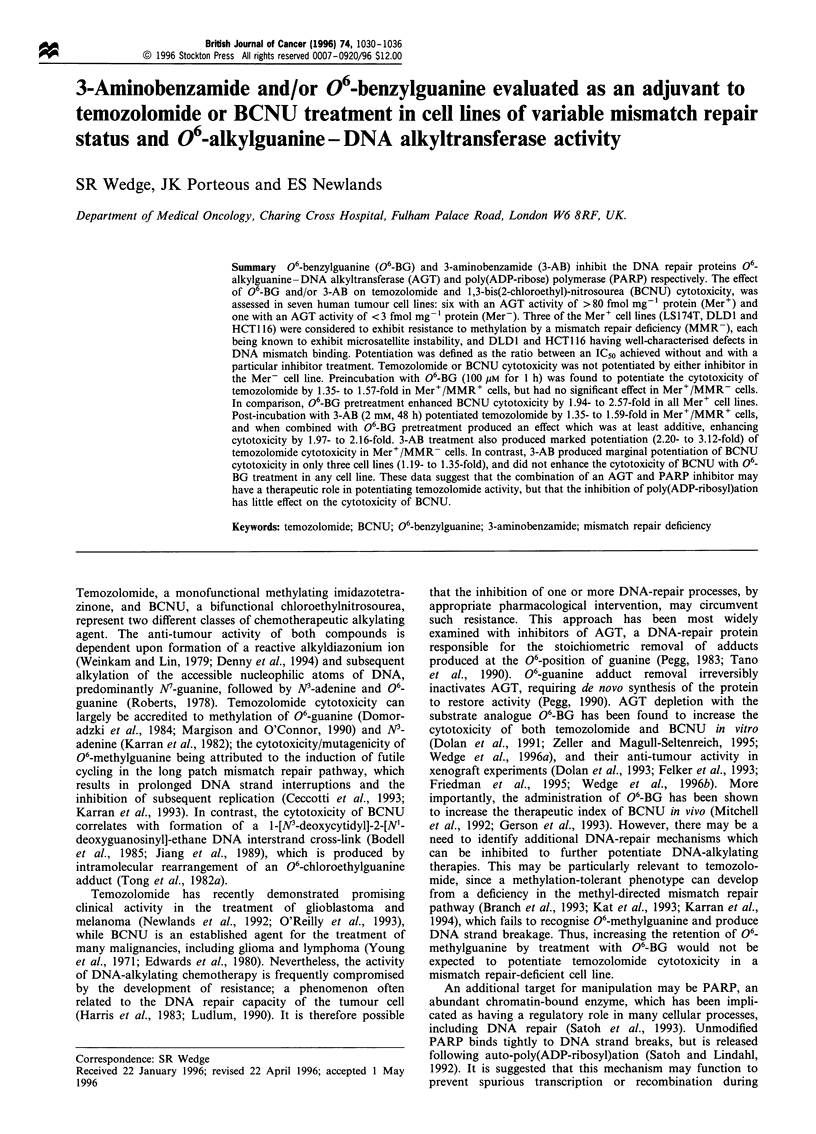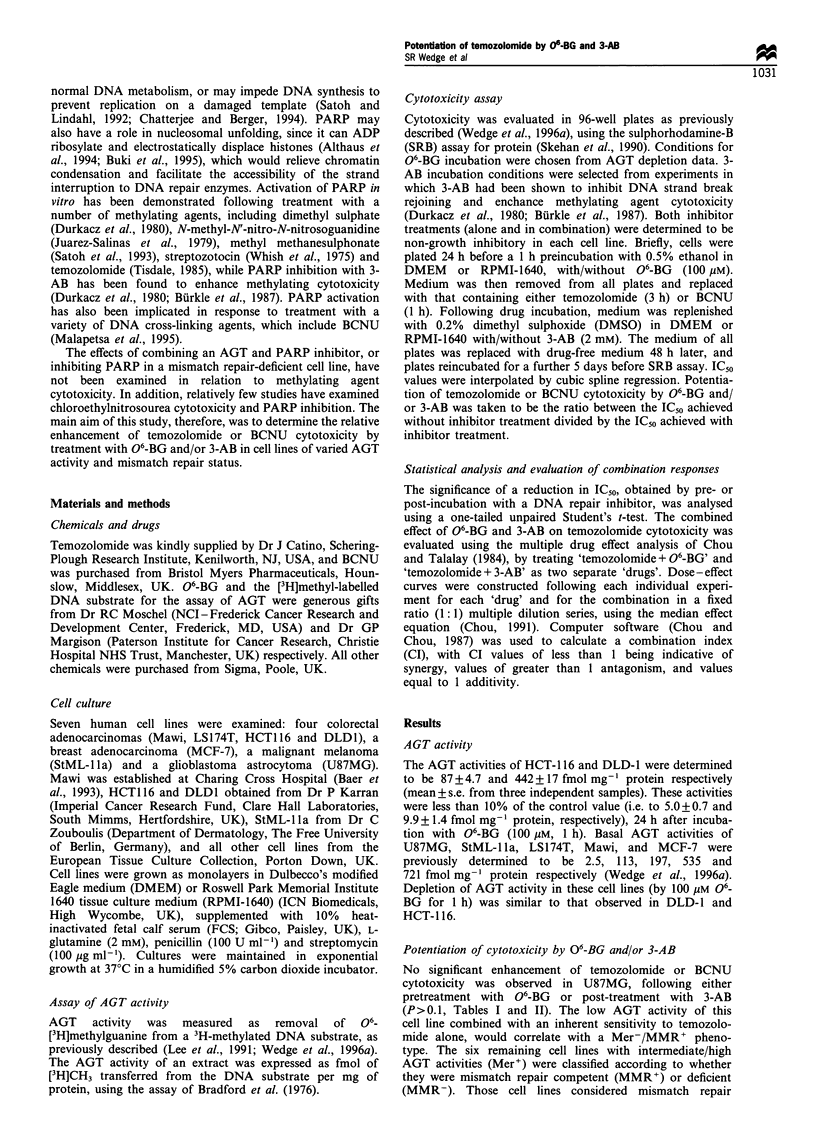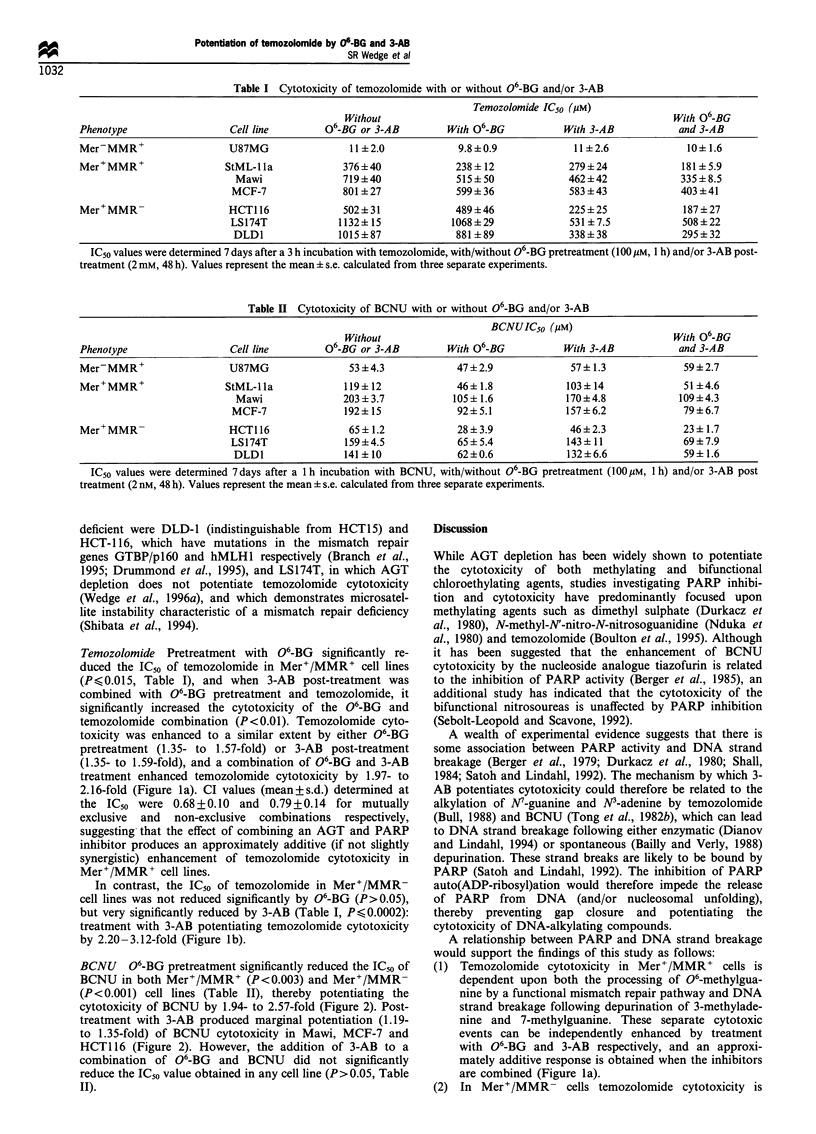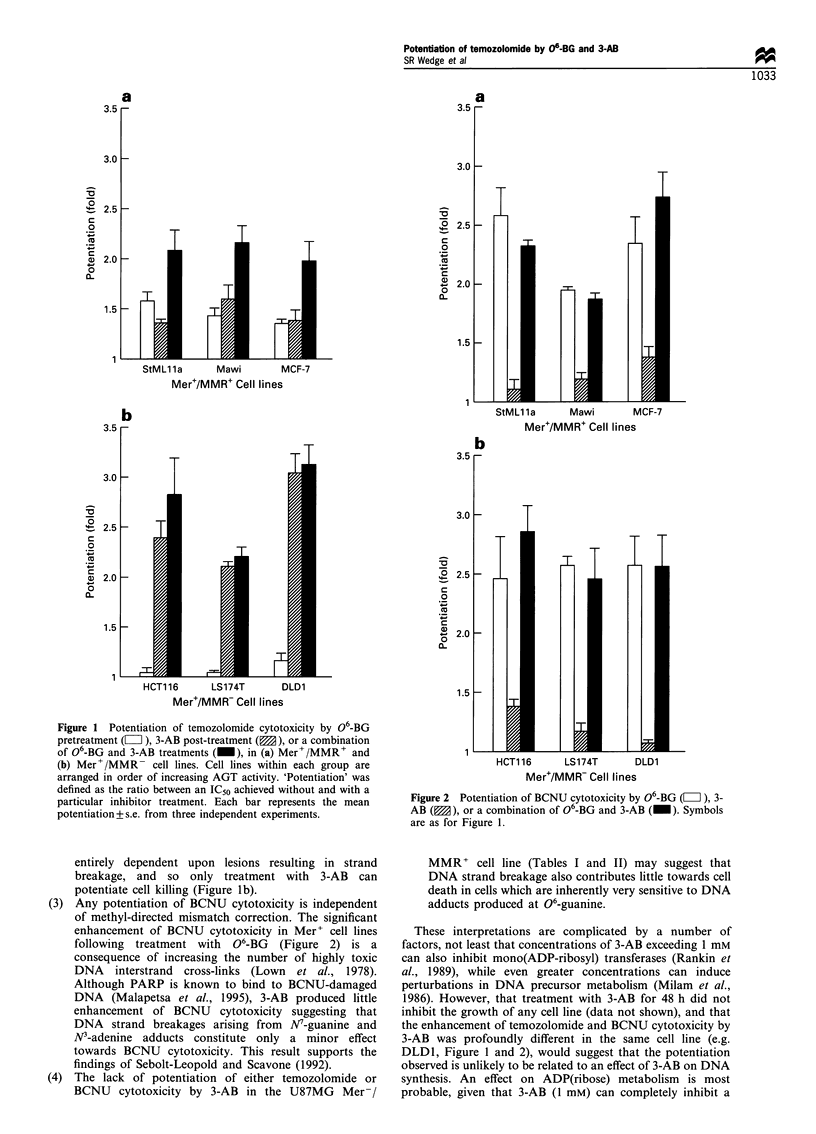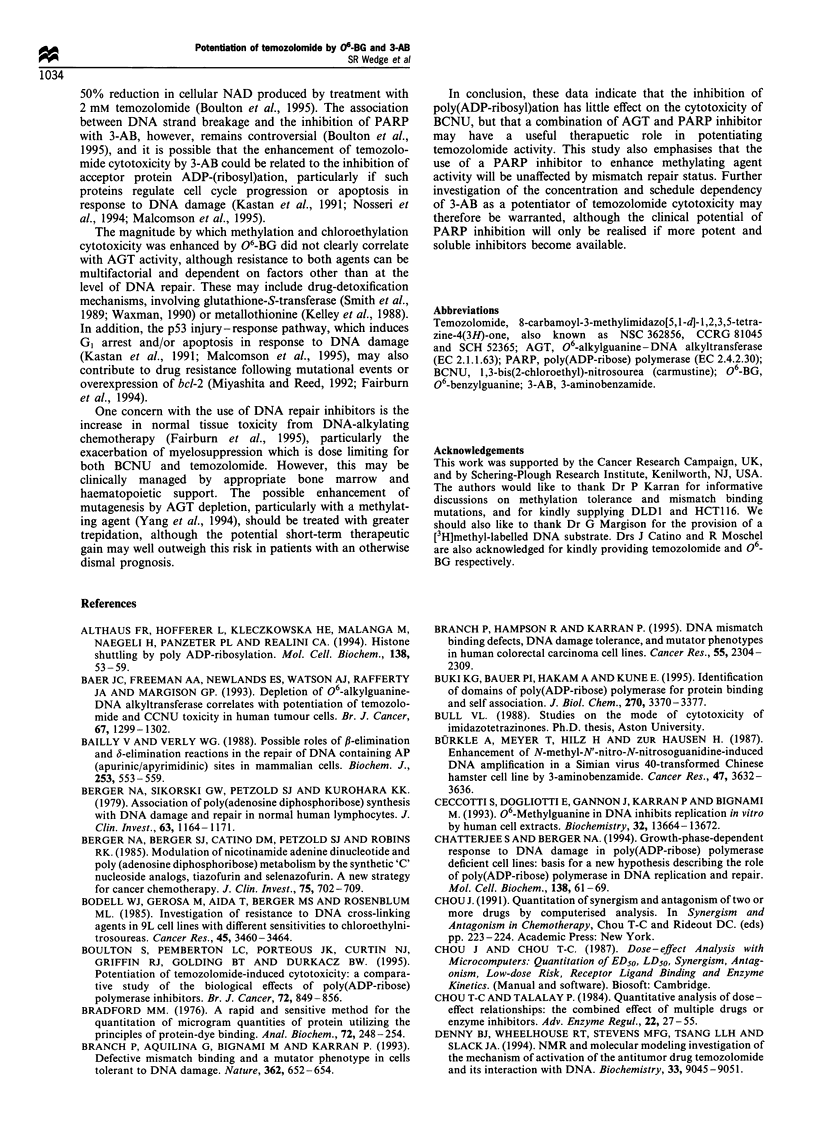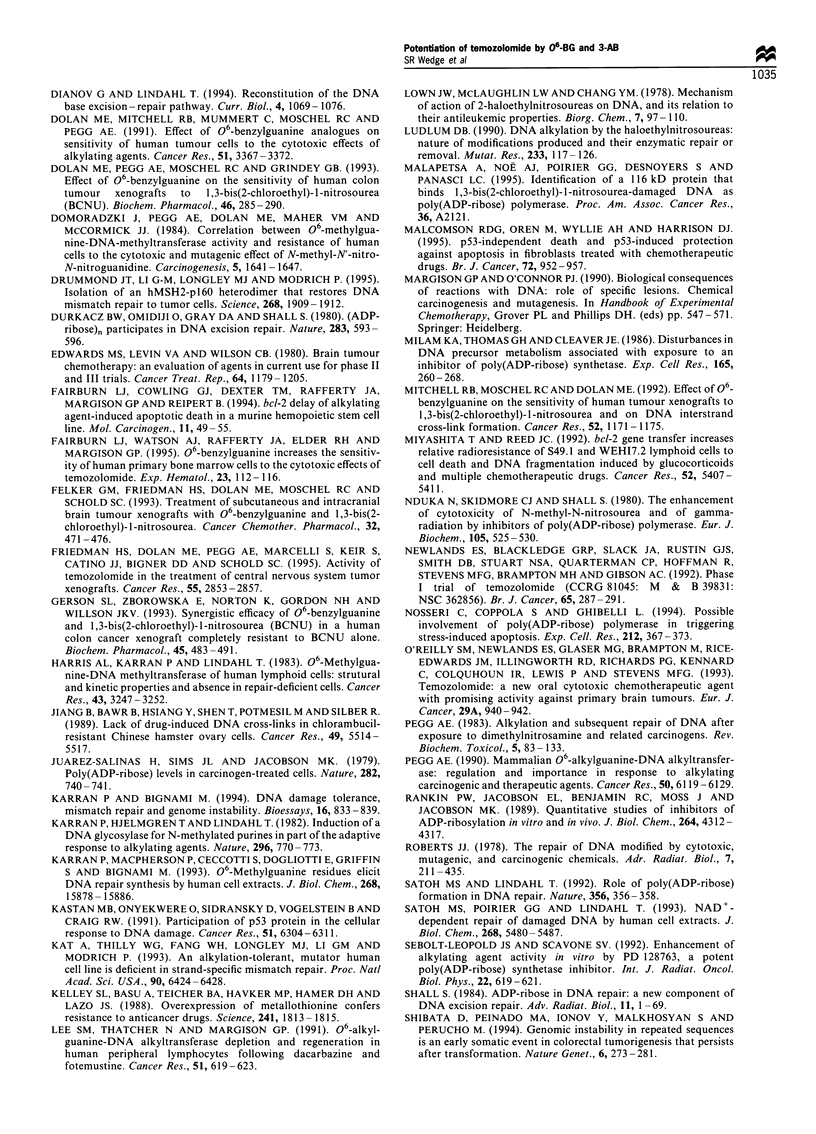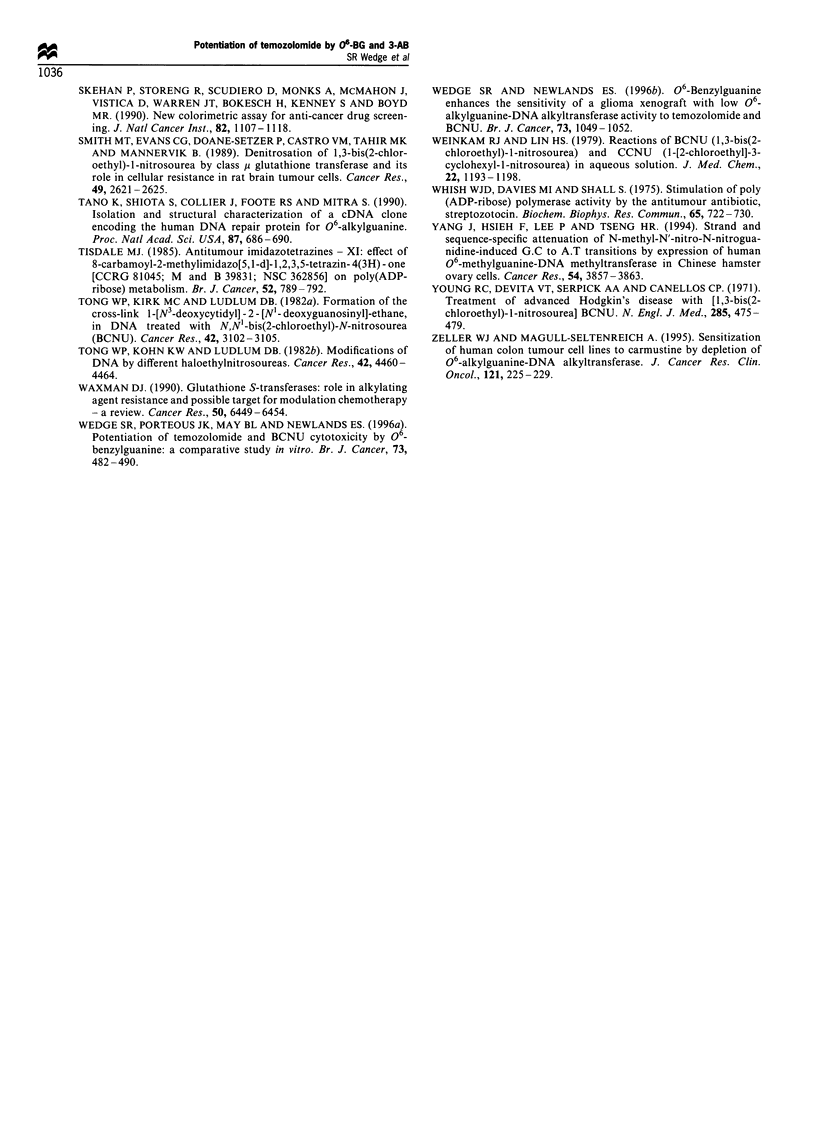# 3-aminobenzamide and/or O6-benzylguanine evaluated as an adjuvant to temozolomide or BCNU treatment in cell lines of variable mismatch repair status and O6-alkylguanine-DNA alkyltransferase activity.

**DOI:** 10.1038/bjc.1996.485

**Published:** 1996-10

**Authors:** S. R. Wedge, J. K. Porteous, E. S. Newlands

**Affiliations:** Department of Medical Oncology, Charing Cross Hospital, London, UK.

## Abstract

O6-benzylguanine (O6-BG) and 3-aminobenzamide (3-AB) inhibit the DNA repair proteins O6-alkylguanine-DNA alkyltransferase (AGT) and poly(ADP-ribose) polymerase (PARP) respectively. The effect of O6-BG and/or 3-AB on temozolomide and 1,3-bis(2-chloroethyl)-nitrosourea (BCNU) cytotoxicity, was assessed in seven human tumour cell lines: six with an AGT activity of > 80 fmol mg-1 protein (Mer+) and one with an AGT activity of < 3 fmol mg-1 protein (Mer-). Three of the Mer+ cell lines (LS174T, DLD1 and HCT116) were considered to exhibit resistance to methylation by a mismatch repair deficiency (MMR-), each being known to exhibit microsatellite instability, and DLD1 and HCT116 having well-characterised defects in DNA mismatch binding. Potentiation was defined as the ratio between an IC50 achieved without and with a particular inhibitor treatment. Temozolomide or BCNU cytotoxicity was not potentiated by either inhibitor in the Mer- cell line. Preincubation with O6-BG (100 microM for 1 h) was found to potentiate the cytotoxicity of temozolomide by 1.35- to 1.57-old in Mer+/MMR+ cells, but had no significant effect in Mer+/MMR- cells. In comparison, O6-BG pretreatment enhanced BCNU cytotoxicity by 1.94- to 2.57-fold in all Mer+ cell lines. Post-incubation with 3-AB (2 mM, 48 h) potentiated temozolomide by 1.35- to 1.59-fold in Mer+/MMR+ cells, and when combined with O6-BG pretreatment produced an effect which was at least additive, enhancing cytotoxicity by 1.97- to 2.16-fold. 3-AB treatment also produced marked potentiation (2.20- to 3.12-fold) of temozolomide cytotoxicity in Mer+/MMR- cells. In contrast, 3-AB produced marginal potentiation of BCNU cytotoxicity in only three cell lines (1.19- to 1.35-fold), and did not enhance the cytotoxicity of BCNU with O6-BG treatment in any cell line. These data suggest that the combination of an AGT and PARP inhibitor may have a therapeutic role in potentiating temozolomide activity, but that the inhibition of poly(ADP-ribosyl)ation has little effect on the cytotoxicity of BCNU.


British Journal of Cancer (1996) 74, 1030-1036
© 1996 Stockton Press All rights reserved 0007-0920/96 $12.00

### 3-Aminobenzamide and/or $O^6$-benzylguanine evaluated as an adjuvant to temozolomide or BCNU treatment in cell lines of variable mismatch repair status and $O^6$-alkylguanine-DNA alkyltransferase activity

SR Wedge, JK Porteous and ES Newlands

*Department of Medical Oncology, Charing Cross Hospital, Fulham Palace Road, London W6 8RF, UK.*

**Summary** $O^6$-benzylguanine ($O^6$-BG) and 3-aminobenzamide (3-AB) inhibit the DNA repair proteins $O^6$-alkylguanine-DNA alkyltransferase (AGT) and poly(ADP-ribose) polymerase (PARP) respectively. The effect of $O^6$-BG and/or 3-AB on temozolomide and 1,3-bis(2-chloroethyl)-nitrosourea (BCNU) cytotoxicity, was assessed in seven human tumour cell lines: six with an AGT activity of >80 fmol $mg^{-1}$ protein ($Mer^+$) and one with an AGT activity of <3 fmol $mg^{-1}$ protein ($Mer^-$). Three of the $Mer^+$ cell lines (LS174T, DLD1 and HCT116) were considered to exhibit resistance to methylation by a mismatch repair deficiency ($MMR^-$), each being known to exhibit microsatellite instability, and DLD1 and HCT116 having well-characterised defects in DNA mismatch binding. Potentiation was defined as the ratio between an $IC_{50}$ achieved without and with a particular inhibitor treatment. Temozolomide or BCNU cytotoxicity was not potentiated by either inhibitor in the $Mer^-$ cell line. Preincubation with $O^6$-BG (100 $\mu$M for 1 h) was found to potentiate the cytotoxicity of temozolomide by 1.35- to 1.57-fold in $Mer^+/MMR^+$ cells, but had no significant effect in $Mer^+/MMR^-$ cells. In comparison, $O^6$-BG pretreatment enhanced BCNU cytotoxicity by 1.94- to 2.57-fold in all $Mer^+$ cell lines. Post-incubation with 3-AB (2 mM, 48 h) potentiated temozolomide by 1.35- to 1.59-fold in $Mer^+/MMR^+$ cells, and when combined with $O^6$-BG pretreatment produced an effect which was at least additive, enhancing cytotoxicity by 1.97- to 2.16-fold. 3-AB treatment also produced marked potentiation (2.20- to 3.12-fold) of temozolomide cytotoxicity in $Mer^+/MMR^-$ cells. In contrast, 3-AB produced marginal potentiation of BCNU cytotoxicity in only three cell lines (1.19- to 1.35-fold), and did not enhance the cytotoxicity of BCNU with $O^6$-BG treatment in any cell line. These data suggest that the combination of an AGT and PARP inhibitor may have a therapeutic role in potentiating temozolomide activity, but that the inhibition of poly(ADP-ribosyl)ation has little effect on the cytotoxicity of BCNU.

**Keywords:** temozolomide; BCNU; $O^6$-benzylguanine; 3-aminobenzamide; mismatch repair deficiency

Temozolomide, a monofunctional methylating imidazotetrazinone, and BCNU, a bifunctional chloroethylnitrosourea, represent two different classes of chemotherapeutic alkylating agent. The anti-tumour activity of both compounds is dependent upon formation of a reactive alkyldiazonium ion (Weinkam and Lin, 1979; Denny *et al.*, 1994) and subsequent alkylation of the accessible nucleophilic atoms of DNA, predominantly $N^7$-guanine, followed by $N^3$-adenine and $O^6$-guanine (Roberts, 1978). Temozolomide cytotoxicity can largely be accredited to methylation of $O^6$-guanine (Domoradzki *et al.*, 1984; Margison and O'Connor, 1990) and $N^3$-adenine (Karran *et al.*, 1982); the cytotoxicity/mutagenicity of $O^6$-methylguanine being attributed to the induction of futile cycling in the long patch mismatch repair pathway, which results in prolonged DNA strand interruptions and the inhibition of subsequent replication (Ceccotti *et al.*, 1993; Karran *et al.*, 1993). In contrast, the cytotoxicity of BCNU correlates with formation of a 1-[$N^3$-deoxycytidyl]-2-[$N^1$-deoxyguanosinyl]-ethane DNA interstrand cross-link (Bodell *et al.*, 1985; Jiang *et al.*, 1989), which is produced by intramolecular rearrangement of an $O^6$-chloroethylguanine adduct (Tong *et al.*, 1982*a*).

Temozolomide has recently demonstrated promising clinical activity in the treatment of glioblastoma and melanoma (Newlands *et al.*, 1992; O'Reilly *et al.*, 1993), while BCNU is an established agent for the treatment of many malignancies, including glioma and lymphoma (Young *et al.*, 1971; Edwards *et al.*, 1980). Nevertheless, the activity of DNA-alkylating chemotherapy is frequently compromised by the development of resistance; a phenomenon often related to the DNA repair capacity of the tumour cell (Harris *et al.*, 1983; Ludlum, 1990). It is therefore possible that the inhibition of one or more DNA-repair processes, by appropriate pharmacological intervention, may circumvent such resistance. This approach has been most widely examined with inhibitors of AGT, a DNA-repair protein responsible for the stoichiometric removal of adducts produced at the $O^6$-position of guanine (Pegg, 1983; Tano *et al.*, 1990). $O^6$-guanine adduct removal irreversibly inactivates AGT, requiring *de novo* synthesis of the protein to restore activity (Pegg, 1990). AGT depletion with the substrate analogue $O^6$-BG has been found to increase the cytotoxicity of both temozolomide and BCNU *in vitro* (Dolan *et al.*, 1991; Zeller and Magull-Seltenreich, 1995; Wedge *et al.*, 1996*a*), and their anti-tumour activity in xenograft experiments (Dolan *et al.*, 1993; Felker *et al.*, 1993; Friedman *et al.*, 1995; Wedge *et al.*, 1996*b*). More importantly, the administration of $O^6$-BG has been shown to increase the therapeutic index of BCNU *in vivo* (Mitchell *et al.*, 1992; Gerson *et al.*, 1993). However, there may be a need to identify additional DNA-repair mechanisms which can be inhibited to further potentiate DNA-alkylating therapies. This may be particularly relevant to temozolomide, since a methylation-tolerant phenotype can develop from a deficiency in the methyl-directed mismatch repair pathway (Branch *et al.*, 1993; Kat *et al.*, 1993; Karran *et al.*, 1994), which fails to recognise $O^6$-methylguanine and produce DNA strand breakage. Thus, increasing the retention of $O^6$-methylguanine by treatment with $O^6$-BG would not be expected to potentiate temozolomide cytotoxicity in a mismatch repair-deficient cell line.

An additional target for manipulation may be PARP, an abundant chromatin-bound enzyme, which has been implicated as having a regulatory role in many cellular processes, including DNA repair (Satoh *et al.*, 1993). Unmodified PARP binds tightly to DNA strand breaks, but is released following auto-poly(ADP-ribosyl)ation (Satoh and Lindahl, 1992). It is suggested that this mechanism may function to prevent spurious transcription or recombination during

Correspondence: SR Wedge
Received 22 January 1996; revised 22 April 1996; accepted 1 May 1996

normal DNA metabolism, or may impede DNA synthesis to prevent replication on a damaged template (Satoh and Lindahl, 1992; Chatterjee and Berger, 1994). PARP may also have a role in nucleosomal unfolding, since it can ADP ribosylate and electrostatically displace histones (Althaus *et al.*, 1994; Buki *et al.*, 1995), which would relieve chromatin condensation and facilitate the accessibility of the strand interruption to DNA repair enzymes. Activation of PARP *in vitro* has been demonstrated following treatment with a number of methylating agents, including dimethyl sulphate (Durkacz *et al.*, 1980), *N*-methyl-*N'*-nitro-*N*-nitrosoguanidine (Juarez-Salinas *et al.*, 1979), methyl methanesulphonate (Satoh *et al.*, 1993), streptozotocin (Whish *et al.*, 1975) and temozolomide (Tisdale, 1985), while PARP inhibition with 3-AB has been found to enhance methylating cytotoxicity (Durkacz *et al.*, 1980; Bürkle *et al.*, 1987). PARP activation has also been implicated in response to treatment with a variety of DNA cross-linking agents, which include BCNU (Malapetsa *et al.*, 1995).

The effects of combining an AGT and PARP inhibitor, or inhibiting PARP in a mismatch repair-deficient cell line, have not been examined in relation to methylating agent cytotoxicity. In addition, relatively few studies have examined chloroethylnitrosourea cytotoxicity and PARP inhibition. The main aim of this study, therefore, was to determine the relative enhancement of temozolomide or BCNU cytotoxicity by treatment with $O^6$-BG and/or 3-AB in cell lines of varied AGT activity and mismatch repair status.

### Materials and methods

### *Chemicals and drugs*

Temozolomide was kindly supplied by Dr J Catino, Schering-Plough Research Institute, Kenilworth, NJ, USA, and BCNU was purchased from Bristol Myers Pharmaceuticals, Hounslow, Middlesex, UK. $O^6$-BG and the [$^3$H]methyl-labelled DNA substrate for the assay of AGT were generous gifts from Dr RC Moschel (NCI–Frederick Cancer Research and Development Center, Frederick, MD, USA) and Dr GP Margison (Paterson Institute for Cancer Research, Christie Hospital NHS Trust, Manchester, UK) respectively. All other chemicals were purchased from Sigma, Poole, UK.

### *Cell culture*

Seven human cell lines were examined: four colorectal adenocarcinomas (Mawi, LS174T, HCT116 and DLD1), a breast adenocarcinoma (MCF-7), a malignant melanoma (StML-11a) and a glioblastoma astrocytoma (U87MG). Mawi was established at Charing Cross Hospital (Baer *et al.*, 1993), HCT116 and DLD1 obtained from Dr P Karran (Imperial Cancer Research Fund, Clare Hall Laboratories, South Mimms, Hertfordshire, UK), StML-11a from Dr C Zouboulis (Department of Dermatology, The Free University of Berlin, Germany), and all other cell lines from the European Tissue Culture Collection, Porton Down, UK. Cell lines were grown as monolayers in Dulbecco's modified Eagle medium (DMEM) or Roswell Park Memorial Institute 1640 tissue culture medium (RPMI-1640) (ICN Biomedicals, High Wycombe, UK), supplemented with 10% heat-inactivated fetal calf serum (FCS; Gibco, Paisley, UK), L-glutamine (2 mM), penicillin (100 U $ml^{-1}$) and streptomycin (100 $\mu g$ $ml^{-1}$). Cultures were maintained in exponential growth at 37°C in a humidified 5% carbon dioxide incubator.

### *Assay of AGT activity*

AGT activity was measured as removal of $O^6$-[$^3$H]methylguanine from a $^3$H-methylated DNA substrate, as previously described (Lee *et al.*, 1991; Wedge *et al.*, 1996*a*). The AGT activity of an extract was expressed as fmol of [$^3$H]$CH_3$ transferred from the DNA substrate per mg of protein, using the assay of Bradford *et al.* (1976).

### *Cytotoxicity assay*

Cytotoxicity was evaluated in 96-well plates as previously described (Wedge *et al.*, 1996*a*), using the sulphorhodamine-B (SRB) assay for protein (Skehan *et al.*, 1990). Conditions for $O^6$-BG incubation were chosen from AGT depletion data. 3-AB incubation conditions were selected from experiments in which 3-AB had been shown to inhibit DNA strand break rejoining and enchance methylating agent cytotoxicity (Durkacz *et al.*, 1980; Bürkle *et al.*, 1987). Both inhibitor treatments (alone and in combination) were determined to be non-growth inhibitory in each cell line. Briefly, cells were plated 24 h before a 1 h preincubation with 0.5% ethanol in DMEM or RPMI-1640, with/without $O^6$-BG (100 $\mu$M). Medium was then removed from all plates and replaced with that containing either temozolomide (3 h) or BCNU (1 h). Following drug incubation, medium was replenished with 0.2% dimethyl sulphoxide (DMSO) in DMEM or RPMI-1640 with/without 3-AB (2 mM). The medium of all plates was replaced with drug-free medium 48 h later, and plates reincubated for a further 5 days before SRB assay. $IC_{50}$ values were interpolated by cubic spline regression. Potentiation of temozolomide or BCNU cytotoxicity by $O^6$-BG and/or 3-AB was taken to be the ratio between the $IC_{50}$ achieved without inhibitor treatment divided by the $IC_{50}$ achieved with inhibitor treatment.

### *Statistical analysis and evaluation of combination responses*

The significance of a reduction in $IC_{50}$, obtained by pre- or post-incubation with a DNA repair inhibitor, was analysed using a one-tailed unpaired Student's *t*-test. The combined effect of $O^6$-BG and 3-AB on temozolomide cytotoxicity was evaluated using the multiple drug effect analysis of Chou and Talalay (1984), by treating 'temozolomide + $O^6$-BG' and 'temozolomide + 3-AB' as two separate 'drugs'. Dose–effect curves were constructed following each individual experiment for each 'drug' and for the combination in a fixed ratio (1 : 1) multiple dilution series, using the median effect equation (Chou, 1991). Computer software (Chou and Chou, 1987) was used to calculate a combination index (CI), with CI values of less than 1 being indicative of synergy, values of greater than 1 antagonism, and values equal to 1 additivity.

### Results

### *AGT activity*

The AGT activities of HCT-116 and DLD-1 were determined to be $87 \pm 4.7$ and $442 \pm 17$ fmol $mg^{-1}$ protein respectively (mean ± s.e. from three independent samples). These activities were less than 10% of the control value (i.e. to $5.0 \pm 0.7$ and $9.9 \pm 1.4$ fmol $mg^{-1}$ protein, respectively), 24 h after incubation with $O^6$-BG (100 $\mu$M, 1 h). Basal AGT activities of U87MG, StML-11a, LS174T, Mawi, and MCF-7 were previously determined to be 2.5, 113, 197, 535 and 721 fmol $mg^{-1}$ protein respectively (Wedge *et al.*, 1996*a*). Depletion of AGT activity in these cell lines (by 100 $\mu$M $O^6$-BG for 1 h) was similar to that observed in DLD-1 and HCT-116.

### *Potentiation of cytotoxicity by $O^6$-BG and/or 3-AB*

No significant enhancement of temozolomide or BCNU cytotoxicity was observed in U87MG, following either pretreatment with $O^6$-BG or post-treatment with 3-AB ($P > 0.1$, Tables I and II). The low AGT activity of this cell line combined with an inherent sensitivity to temozolomide alone, would correlate with a $Mer^-/MMR^+$ phenotype. The six remaining cell lines with intermediate/high AGT activities ($Mer^+$) were classified according to whether they were mismatch repair competent ($MMR^+$) or deficient ($MMR^-$). Those cell lines considered mismatch repair

**Table I** Cytotoxicity of temozolomide with or without $O^6$-BG and/or 3-AB

| *Phenotype* | *Cell line* | *Temozolomide $IC_{50}$ (μM)* Without $O^6$-BG or 3-AB | With $O^6$-BG | With 3-AB | With $O^6$-BG and 3-AB |
|---|---|---|---|---|---|
| $Mer^-MMR^+$ | U87MG | 11±2.0 | 9.8±0.9 | 11±2.6 | 10±1.6 |
| $Mer^+MMR^+$ | StML-11a | 376±40 | 238±12 | 279±24 | 181±5.9 |
| | Mawi | 719±40 | 515±50 | 462±42 | 335±8.5 |
| | MCF-7 | 801±27 | 599±36 | 583±43 | 403±41 |
| $Mer^+MMR^-$ | HCT116 | 502±31 | 489±46 | 225±25 | 187±27 |
| | LS174T | 1132±15 | 1068±29 | 531±7.5 | 508±22 |
| | DLD1 | 1015±87 | 881±89 | 338±38 | 295±32 |

$IC_{50}$ values were determined 7 days after a 3 h incubation with temozolomide, with/without $O^6$-BG pretreatment (100 μM, 1 h) and/or 3-AB post-treatment (2 mM, 48 h). Values represent the mean ± s.e. calculated from three separate experiments.

**Table II** Cytotoxicity of BCNU with or without $O^6$-BG and/or 3-AB

| *Phenotype* | *Cell line* | *BCNU $IC_{50}$ (μM)* Without $O^6$-BG or 3-AB | With $O^6$-BG | With 3-AB | With $O^6$-BG and 3-AB |
|---|---|---|---|---|---|
| $Mer^-MMR^+$ | U87MG | 53±4.3 | 47±2.9 | 57±1.3 | 59±2.7 |
| $Mer^+MMR^+$ | StML-11a | 119±12 | 46±1.8 | 103±14 | 51±4.6 |
| | Mawi | 203±3.7 | 105±1.6 | 170±4.8 | 109±4.3 |
| | MCF-7 | 192±15 | 92±5.1 | 157±6.2 | 79±6.7 |
| $Mer^+MMR^-$ | HCT116 | 65±1.2 | 28±3.9 | 46±2.3 | 23±1.7 |
| | LS174T | 159±4.5 | 65±5.4 | 143±11 | 69±7.9 |
| | DLD1 | 141±10 | 62±0.6 | 132±6.6 | 59±1.6 |

$IC_{50}$ values were determined 7 days after a 1 h incubation with BCNU, with/without $O^6$-BG pretreatment (100 μM, 1 h) and/or 3-AB post treatment (2 nM, 48 h). Values represent the mean ± s.e. calculated from three separate experiments.

deficient were DLD-1 (indistinguishable from HCT15) and HCT-116, which have mutations in the mismatch repair genes GTBP/p160 and hMLH1 respectively (Branch *et al.*, 1995; Drummond *et al.*, 1995), and LS174T, in which AGT depletion does not potentiate temozolomide cytotoxicity (Wedge *et al.*, 1996*a*), and which demonstrates microsatellite instability characteristic of a mismatch repair deficiency (Shibata *et al.*, 1994).

*Temozolomide* Pretreatment with $O^6$-BG significantly reduced the $IC_{50}$ of temozolomide in $Mer^+/MMR^+$ cell lines ($P \leqslant 0.015$, Table I), and when 3-AB post-treatment was combined with $O^6$-BG pretreatment and temozolomide, it significantly increased the cytotoxicity of the $O^6$-BG and temozolomide combination ($P < 0.01$). Temozolomide cytotoxicity was enhanced to a similar extent by either $O^6$-BG pretreatment (1.35- to 1.57-fold) or 3-AB post-treatment (1.35- to 1.59-fold), and a combination of $O^6$-BG and 3-AB treatment enhanced temozolomide cytotoxicity by 1.97- to 2.16-fold (Figure 1a). CI values (mean±s.d.) determined at the $IC_{50}$ were 0.68±0.10 and 0.79±0.14 for mutually exclusive and non-exclusive combinations respectively, suggesting that the effect of combining an AGT and PARP inhibitor produces an approximately additive (if not slightly synergistic) enhancement of temozolomide cytotoxicity in $Mer^+/MMR^+$ cell lines.

In contrast, the $IC_{50}$ of temozolomide in $Mer^+/MMR^-$ cell lines was not reduced significantly by $O^6$-BG ($P > 0.05$), but very significantly reduced by 3-AB (Table I, $P \leqslant 0.0002$): treatment with 3-AB potentiating temozolomide cytotoxicity by 2.20–3.12-fold (Figure 1b).

*BCNU* $O^6$-BG pretreatment significantly reduced the $IC_{50}$ of BCNU in both $Mer^+/MMR^+$ ($P < 0.003$) and $Mer^+/MMR^-$ ($P < 0.001$) cell lines (Table II), thereby potentiating the cytotoxicity of BCNU by 1.94- to 2.57-fold (Figure 2). Post-treatment with 3-AB produced marginal potentiation (1.19- to 1.35-fold) of BCNU cytotoxicity in Mawi, MCF-7 and HCT116 (Figure 2). However, the addition of 3-AB to a combination of $O^6$-BG and BCNU did not significantly reduce the $IC_{50}$ value obtained in any cell line ($P > 0.05$, Table II).

### Discussion

While AGT depletion has been widely shown to potentiate the cytotoxicity of both methylating and bifunctional chloroethylating agents, studies investigating PARP inhibition and cytotoxicity have predominantly focused upon methylating agents such as dimethyl sulphate (Durkacz *et al.*, 1980), *N*-methyl-*N'*-nitro-*N*-nitrosoguanidine (Nduka *et al.*, 1980) and temozolomide (Boulton *et al.*, 1995). Although it has been suggested that the enhancement of BCNU cytotoxicity by the nucleoside analogue tiazofurin is related to the inhibition of PARP activity (Berger *et al.*, 1985), an additional study has indicated that the cytotoxicity of the bifunctional nitrosoureas is unaffected by PARP inhibition (Sebolt-Leopold and Scavone, 1992).

A wealth of experimental evidence suggests that there is some association between PARP activity and DNA strand breakage (Berger *et al.*, 1979; Durkacz *et al.*, 1980; Shall, 1984; Satoh and Lindahl, 1992). The mechanism by which 3-AB potentiates cytotoxicity could therefore be related to the alkylation of $N^7$-guanine and $N^3$-adenine by temozolomide (Bull, 1988) and BCNU (Tong *et al.*, 1982*b*), which can lead to DNA strand breakage following either enzymatic (Dianov and Lindahl, 1994) or spontaneous (Bailly and Verly, 1988) depurination. These strand breaks are likely to be bound by PARP (Satoh and Lindahl, 1992). The inhibition of PARP auto(ADP-ribosyl)ation would therefore impede the release of PARP from DNA (and/or nucleosomal unfolding), thereby preventing gap closure and potentiating the cytotoxicity of DNA-alkylating compounds.

A relationship between PARP and DNA strand breakage would support the findings of this study as follows:

(1) Temozolomide cytotoxicity in $Mer^+/MMR^+$ cells is dependent upon both the processing of $O^6$-methylguanine by a functional mismatch repair pathway and DNA strand breakage following depurination of 3-methyladenine and 7-methylguanine. These separate cytotoxic events can be independently enhanced by treatment with $O^6$-BG and 3-AB respectively, and an approximately additive response is obtained when the inhibitors are combined (Figure 1a).

(2) In $Mer^+/MMR^-$ cells temozolomide cytotoxicity is

**Figure 1** Potentiation of temozolomide cytotoxicity by $O^6$-BG pretreatment (□), 3-AB post-treatment (▨), or a combination of $O^6$-BG and 3-AB treatments (■), in (**a**) $Mer^+/MMR^+$ and (**b**) $Mer^+/MMR^-$ cell lines. Cell lines within each group are arranged in order of increasing AGT activity. 'Potentiation' was defined as the ratio between an $IC_{50}$ achieved without and with a particular inhibitor treatment. Each bar represents the mean potentiation ± s.e. from three independent experiments.

**Figure 2** Potentiation of BCNU cytotoxicity by $O^6$-BG (□), 3-AB (▨), or a combination of $O^6$-BG and 3-AB (■). Symbols are as for Figure 1.

entirely dependent upon lesions resulting in strand breakage, and so only treatment with 3-AB can potentiate cell killing (Figure 1b).

(3) Any potentiation of BCNU cytotoxicity is independent of methyl-directed mismatch correction. The significant enhancement of BCNU cytotoxicity in $Mer^+$ cell lines following treatment with $O^6$-BG (Figure 2) is a consequence of increasing the number of highly toxic DNA interstrand cross-links (Lown *et al.*, 1978). Although PARP is known to bind to BCNU-damaged DNA (Malapetsa *et al.*, 1995), 3-AB produced little enhancement of BCNU cytotoxicity suggesting that DNA strand breakages arising from $N^7$-guanine and $N^3$-adenine adducts constitute only a minor effect towards BCNU cytotoxicity. This result supports the findings of Sebolt-Leopold and Scavone (1992).

(4) The lack of potentiation of either temozolomide or BCNU cytotoxicity by 3-AB in the U87MG $Mer^-/MMR^+$ cell line (Tables I and II) may suggest that DNA strand breakage also contributes little towards cell death in cells which are inherently very sensitive to DNA adducts produced at $O^6$-guanine.

These interpretations are complicated by a number of factors, not least that concentrations of 3-AB exceeding 1 mM can also inhibit mono(ADP-ribosyl) transferases (Rankin *et al.*, 1989), while even greater concentrations can induce perturbations in DNA precursor metabolism (Milam *et al.*, 1986). However, that treatment with 3-AB for 48 h did not inhibit the growth of any cell line (data not shown), and that the enhancement of temozolomide and BCNU cytotoxicity by 3-AB was profoundly different in the same cell line (e.g. DLD1, Figure 1 and 2), would suggest that the potentiation observed is unlikely to be related to an effect of 3-AB on DNA synthesis. An effect on ADP(ribose) metabolism is most probable, given that 3-AB (1 mM) can completely inhibit a

50% reduction in cellular NAD produced by treatment with 2 mM temozolomide (Boulton et al., 1995). The association between DNA strand breakage and the inhibition of PARP with 3-AB, however, remains controversial (Boulton et al., 1995), and it is possible that the enhancement of temozolomide cytotoxicity by 3-AB could be related to the inhibition of acceptor protein ADP-(ribosyl)ation, particularly if such proteins regulate cell cycle progression or apoptosis in response to DNA damage (Kastan et al., 1991; Nosseri et al., 1994; Malcomson et al., 1995).

The magnitude by which methylation and chloroethylation cytotoxicity was enhanced by $O^6$-BG did not clearly correlate with AGT activity, although resistance to both agents can be multifactorial and dependent on factors other than at the level of DNA repair. These may include drug-detoxification mechanisms, involving glutathione-*S*-transferase (Smith et al., 1989; Waxman, 1990) or metallothionine (Kelley et al., 1988). In addition, the p53 injury–response pathway, which induces $G_1$ arrest and/or apoptosis in response to DNA damage (Kastan et al., 1991; Malcomson et al., 1995), may also contribute to drug resistance following mutational events or overexpression of *bcl*-2 (Miyashita and Reed, 1992; Fairburn et al., 1994).

One concern with the use of DNA repair inhibitors is the increase in normal tissue toxicity from DNA-alkylating chemotherapy (Fairburn et al., 1995), particularly the exacerbation of myelosuppression which is dose limiting for both BCNU and temozolomide. However, this may be clinically managed by appropriate bone marrow and haematopoietic support. The possible enhancement of mutagenesis by AGT depletion, particularly with a methylating agent (Yang et al., 1994), should be treated with greater trepidation, although the potential short-term therapeutic gain may well outweigh this risk in patients with an otherwise dismal prognosis.

In conclusion, these data indicate that the inhibition of poly(ADP-ribosyl)ation has little effect on the cytotoxicity of BCNU, but that a combination of AGT and PARP inhibitor may have a useful therapuetic role in potentiating temozolomide activity. This study also emphasises that the use of a PARP inhibitor to enhance methylating agent activity will be unaffected by mismatch repair status. Further investigation of the concentration and schedule dependency of 3-AB as a potentiator of temozolomide cytotoxicity may therefore be warranted, although the clinical potential of PARP inhibition will only be realised if more potent and soluble inhibitors become available.

### Abbreviations

Temozolomide, 8-carbamoyl-3-methylimidazo[5,1-*d*]-1,2,3,5-tetrazine-4(3*H*)-one, also known as NSC 362856, CCRG 81045 and SCH 52365; AGT, $O^6$-alkylguanine–DNA alkyltransferase (EC 2.1.1.63); PARP, poly(ADP-ribose) polymerase (EC 2.4.2.30); BCNU, 1,3-bis(2-chloroethyl)-nitrosourea (carmustine); $O^6$-BG, $O^6$-benzylguanine; 3-AB, 3-aminobenzamide.

### Acknowledgements

This work was supported by the Cancer Research Campaign, UK, and by Schering-Plough Research Institute, Kenilworth, NJ, USA. The authors would like to thank Dr P Karran for informative discussions on methylation tolerance and mismatch binding mutations, and for kindly supplying DLD1 and HCT116. We should also like to thank Dr G Margison for the provision of a [$^3$H]methyl-labelled DNA substrate. Drs J Catino and R Moschel are also acknowledged for kindly providing temozolomide and $O^6$-BG respectively.

### References

ALTHAUS FR, HOFFERER L, KLECZKOWSKA HE, MALANGA M, NAEGELI H, PANZETER PL AND REALINI CA. (1994). Histone shuttling by poly ADP-ribosylation. *Mol. Cell. Biochem.*, **138**, 53–59.

BAER JC, FREEMAN AA, NEWLANDS ES, WATSON AJ, RAFFERTY JA AND MARGISON GP. (1993). Depletion of $O^6$-alkylguanine-DNA alkyltransferase correlates with potentiation of temozolomide and CCNU toxicity in human tumour cells. *Br. J. Cancer*, **67**, 1299–1302.

BAILLY V AND VERLY WG. (1988). Possible roles of $\beta$-elimination and $\delta$-elimination reactions in the repair of DNA containing AP (apurinic/apyrimidinic) sites in mammalian cells. *Biochem. J.*, **253**, 553–559.

BERGER NA, SIKORSKI GW, PETZOLD SJ AND KUROHARA KK. (1979). Association of poly(adenosine diphosphoribose) synthesis with DNA damage and repair in normal human lymphocytes. *J. Clin. Invest.*, **63**, 1164–1171.

BERGER NA, BERGER SJ, CATINO DM, PETZOLD SJ AND ROBINS RK. (1985). Modulation of nicotinamide adenine dinucleotide and poly (adenosine diphosphoribose) metabolism by the synthetic 'C' nucleoside analogs, tiazofurin and selenazofurin. A new strategy for cancer chemotherapy. *J. Clin. Invest.*, **75**, 702–709.

BODELL WJ, GEROSA M, AIDA T, BERGER MS AND ROSENBLUM ML. (1985). Investigation of resistance to DNA cross-linking agents in 9L cell lines with different sensitivities to chloroethylnitrosoureas. *Cancer Res.*, **45**, 3460–3464.

BOULTON S, PEMBERTON LC, PORTEOUS JK, CURTIN NJ, GRIFFIN RJ, GOLDING BT AND DURKACZ BW. (1995). Potentiation of temozolomide-induced cytotoxicity: a comparative study of the biological effects of poly(ADP-ribose) polymerase inhibitors. *Br. J. Cancer*, **72**, 849–856.

BRADFORD MM. (1976). A rapid and sensitive method for the quantitation of microgram quantities of protein utilizing the principles of protein-dye binding. *Anal. Biochem.*, **72**, 248–254.

BRANCH P, AQUILINA G, BIGNAMI M AND KARRAN P. (1993). Defective mismatch binding and a mutator phenotype in cells tolerant to DNA damage. *Nature*, **362**, 652–654.

BRANCH P, HAMPSON R AND KARRAN P. (1995). DNA mismatch binding defects, DNA damage tolerance, and mutator phenotypes in human colorectal carcinoma cell lines. *Cancer Res.*, **55**, 2304–2309.

BUKI KG, BAUER PI, HAKAM A AND KUNE E. (1995). Identification of domains of poly(ADP-ribose) polymerase for protein binding and self association. *J. Biol. Chem.*, **270**, 3370–3377.

BULL VL. (1988). Studies on the mode of cytotoxicity of imidazotetrazinones. Ph.D. thesis, Aston University.

BÜRKLE A, MEYER T, HILZ H AND ZUR HAUSEN H. (1987). Enhancement of *N*-methyl-*N'*-nitro-*N*-nitrosoguanidine-induced DNA amplification in a Simian virus 40-transformed Chinese hamster cell line by 3-aminobenzamide. *Cancer Res.*, **47**, 3632–3636.

CECCOTTI S, DOGLIOTTI E, GANNON J, KARRAN P AND BIGNAMI M. (1993). $O^6$-Methylguanine in DNA inhibits replication *in vitro* by human cell extracts. *Biochemistry*, **32**, 13664–13672.

CHATTERJEE S AND BERGER NA. (1994). Growth-phase-dependent response to DNA damage in poly(ADP-ribose) polymerase deficient cell lines: basis for a new hypothesis describing the role of poly(ADP-ribose) polymerase in DNA replication and repair. *Mol. Cell. Biochem.*, **138**, 61–69.

CHOU J. (1991). Quantitation of synergism and antagonism of two or more drugs by computerised analysis. In *Synergism and Antagonism in Chemotherapy*, Chou T-C and Rideout DC. (eds) pp. 223–224. Academic Press: New York.

CHOU J AND CHOU T-C. (1987). *Dose–effect Analysis with Microcomputers: Quantitation of $ED_{50}$, $LD_{50}$, Synergism, Antagonism, Low-dose Risk, Receptor Ligand Binding and Enzyme Kinetics.* (Manual and software). Biosoft: Cambridge.

CHOU T-C AND TALALAY P. (1984). Quantitative analysis of dose–effect relationships: the combined effect of multiple drugs or enzyme inhibitors. *Adv. Enzyme Regul.*, **22**, 27–55.

DENNY BJ, WHEELHOUSE RT, STEVENS MFG, TSANG LLH AND SLACK JA. (1994). NMR and molecular modeling investigation of the mechanism of activation of the antitumor drug temozolomide and its interaction with DNA. *Biochemistry*, **33**, 9045–9051.

DIANOV G AND LINDAHL T. (1994). Reconstitution of the DNA base excision-repair pathway. *Curr. Biol.*, **4**, 1069-1076.

DOLAN ME, MITCHELL RB, MUMMERT C, MOSCHEL RC AND PEGG AE. (1991). Effect of $O^6$-benzylguanine analogues on sensitivity of human tumour cells to the cytotoxic effects of alkylating agents. *Cancer Res.*, **51**, 3367-3372.

DOLAN ME, PEGG AE, MOSCHEL RC AND GRINDEY GB. (1993). Effect of $O^6$-benzylguanine on the sensitivity of human colon tumour xenografts to 1,3-bis(2-chloroethyl)-1-nitrosourea (BCNU). *Biochem. Pharmacol.*, **46**, 285-290.

DOMORADZKI J, PEGG AE, DOLAN ME, MAHER VM AND McCORMICK JJ. (1984). Correlation between $O^6$-methylguanine-DNA-methyltransferase activity and resistance of human cells to the cytotoxic and mutagenic effect of *N*-methyl-*N'*-nitro-*N*-nitroguanidine. *Carcinogenesis*, **5**, 1641-1647.

DRUMMOND JT, LI G-M, LONGLEY MJ AND MODRICH P. (1995). Isolation of an hMSH2-p160 heterodimer that restores DNA mismatch repair to tumor cells. *Science*, **268**, 1909-1912.

DURKACZ BW, OMIDIJI O, GRAY DA AND SHALL S. (1980). (ADP-ribose)$_n$ participates in DNA excision repair. *Nature*, **283**, 593-596.

EDWARDS MS, LEVIN VA AND WILSON CB. (1980). Brain tumour chemotherapy: an evaluation of agents in current use for phase II and III trials. *Cancer Treat. Rep.*, **64**, 1179-1205.

FAIRBURN LJ, COWLING GJ, DEXTER TM, RAFFERTY JA, MARGISON GP AND REIPERT B. (1994). *bcl*-2 delay of alkylating agent-induced apoptotic death in a murine hemopoietic stem cell line. *Mol. Carcinogen.*, **11**, 49-55.

FAIRBURN LJ, WATSON AJ, RAFFERTY JA, ELDER RH AND MARGISON GP. (1995). $O^6$-benzylguanine increases the sensitivity of human primary bone marrow cells to the cytotoxic effects of temozolomide. *Exp. Hematol.*, **23**, 112-116.

FELKER GM, FRIEDMAN HS, DOLAN ME, MOSCHEL RC AND SCHOLD SC. (1993). Treatment of subcutaneous and intracranial brain tumour xenografts with $O^6$-benzylguanine and 1,3-bis(2-chloroethyl)-1-nitrosourea. *Cancer Chemother. Pharmacol.*, **32**, 471-476.

FRIEDMAN HS, DOLAN ME, PEGG AE, MARCELLI S, KEIR S, CATINO JJ, BIGNER DD AND SCHOLD SC. (1995). Activity of temozolomide in the treatment of central nervous system tumor xenografts. *Cancer Res.*, **55**, 2853-2857.

GERSON SL, ZBOROWSKA E, NORTON K, GORDON NH AND WILLSON JKV. (1993). Synergistic efficacy of $O^6$-benzylguanine and 1,3-bis(2-chloroethyl)-1-nitrosourea (BCNU) in a human colon cancer xenograft completely resistant to BCNU alone. *Biochem. Pharmacol.*, **45**, 483-491.

HARRIS AL, KARRAN P AND LINDAHL T. (1983). $O^6$-Methylguanine-DNA methyltransferase of human lymphoid cells: strutural and kinetic properties and absence in repair-deficient cells. *Cancer Res.*, **43**, 3247-3252.

JIANG B, BAWR B, HSIANG Y, SHEN T, POTMESIL M AND SILBER R. (1989). Lack of drug-induced DNA cross-links in chlorambucil-resistant Chinese hamster ovary cells. *Cancer Res.*, **49**, 5514-5517.

JUAREZ-SALINAS H, SIMS JL AND JACOBSON MK. (1979). Poly(ADP-ribose) levels in carcinogen-treated cells. *Nature*, **282**, 740-741.

KARRAN P AND BIGNAMI M. (1994). DNA damage tolerance, mismatch repair and genome instability. *Bioessays*, **16**, 833-839.

KARRAN P, HJELMGREN T AND LINDAHL T. (1982). Induction of a DNA glycosylase for N-methylated purines in part of the adaptive response to alkylating agents. *Nature*, **296**, 770-773.

KARRAN P, MACPHERSON P, CECCOTTI S, DOGLIOTTI E, GRIFFIN S AND BIGNAMI M. (1993). $O^6$-Methylguanine residues elicit DNA repair synthesis by human cell extracts. *J. Biol. Chem.*, **268**, 15878-15886.

KASTAN MB, ONYEKWERE O, SIDRANSKY D, VOGELSTEIN B AND CRAIG RW. (1991). Participation of p53 protein in the cellular response to DNA damage. *Cancer Res.*, **51**, 6304-6311.

KAT A, THILLY WG, FANG WH, LONGLEY MJ, LI GM AND MODRICH P. (1993). An alkylation-tolerant, mutator human cell line is deficient in strand-specific mismatch repair. *Proc. Natl Acad. Sci. USA.*, **90**, 6424-6428.

KELLEY SL, BASU A, TEICHER BA, HAVKER MP, HAMER DH AND LAZO JS. (1988). Overexpression of metallothionine confers resistance to anticancer drugs. *Science*, **241**, 1813-1815.

LEE SM, THATCHER N AND MARGISON GP. (1991). $O^6$-alkylguanine-DNA alkyltransferase depletion and regeneration in human peripheral lymphocytes following dacarbazine and fotemustine. *Cancer Res.*, **51**, 619-623.

LOWN JW, McLAUGHLIN LW AND CHANG YM. (1978). Mechanism of action of 2-haloethylnitrosoureas on DNA, and its relation to their antileukemic properties. *Biorg. Chem.*, **7**, 97-110.

LUDLUM DB. (1990). DNA alkylation by the haloethylnitrosoureas: nature of modifications produced and their enzymatic repair or removal. *Mutat. Res.*, **233**, 117-126.

MALAPETSA A, NOË AJ, POIRIER GG, DESNOYERS S AND PANASCI LC. (1995). Identification of a 116 kD protein that binds 1,3-bis(2-chloroethyl)-1-nitrosourea-damaged DNA as poly(ADP-ribose) polymerase. *Proc. Am. Assoc. Cancer Res.*, **36**, A2121.

MALCOMSON RDG, OREN M, WYLLIE AH AND HARRISON DJ. (1995). p53-independent death and p53-induced protection against apoptosis in fibroblasts treated with chemotherapeutic drugs. *Br. J. Cancer*, **72**, 952-957.

MARGISON GP AND O'CONNOR PJ. (1990). Biological consequences of reactions with DNA: role of specific lesions. Chemical carcinogenesis and mutagenesis. In *Handbook of Experimental Chemotherapy*, Grover PL and Phillips DH. (eds) pp. 547-571. Springer: Heidelberg.

MILAM KA, THOMAS GH AND CLEAVER JE. (1986). Disturbances in DNA precursor metabolism associated with exposure to an inhibitor of poly(ADP-ribose) synthetase. *Exp. Cell Res.*, **165**, 260-268.

MITCHELL RB, MOSCHEL RC AND DOLAN ME. (1992). Effect of $O^6$-benzylguanine on the sensitivity of human tumour xenografts to 1,3-bis(2-chloroethyl)-1-nitrosourea and on DNA interstrand cross-link formation. *Cancer Res.*, **52**, 1171-1175.

MIYASHITA T AND REED JC. (1992). *bcl*-2 gene transfer increases relative radioresistance of S49.1 and WEHI7.2 lymphoid cells to cell death and DNA fragmentation induced by glucocorticoids and multiple chemotherapeutic drugs. *Cancer Res.*, **52**, 5407-5411.

NDUKA N, SKIDMORE CJ AND SHALL S. (1980). The enhancement of cytotoxicity of N-methyl-N-nitrosourea and of gamma-radiation by inhibitors of poly(ADP-ribose) polymerase. *Eur. J. Biochem.*, **105**, 525-530.

NEWLANDS ES, BLACKLEDGE GRP, SLACK JA, RUSTIN GJS, SMITH DB, STUART NSA, QUARTERMAN CP, HOFFMAN R, STEVENS MFG, BRAMPTON MH AND GIBSON AC. (1992). Phase I trial of temozolomide (CCRG 81045: M & B 39831: NSC 362856). *Br. J. Cancer*, **65**, 287-291.

NOSSERI C, COPPOLA S AND GHIBELLI L. (1994). Possible involvement of poly(ADP-ribose) polymerase in triggering stress-induced apoptosis. *Exp. Cell. Res.*, **212**, 367-373.

O'REILLY SM, NEWLANDS ES, GLASER MG, BRAMPTON M, RICE-EDWARDS JM, ILLINGWORTH RD, RICHARDS PG, KENNARD C, COLQUHOUN IR, LEWIS P AND STEVENS MFG. (1993). Temozolomide: a new oral cytotoxic chemotherapeutic agent with promising activity against primary brain tumours. *Eur. J. Cancer*, **29A**, 940-942.

PEGG AE. (1983). Alkylation and subsequent repair of DNA after exposure to dimethylnitrosamine and related carcinogens. *Rev. Biochem. Toxicol.*, **5**, 83-133.

PEGG AE. (1990). Mammalian $O^6$-alkylguanine-DNA alkyltransferase: regulation and importance in response to alkylating carcinogenic and therapeutic agents. *Cancer Res.*, **50**, 6119-6129.

RANKIN PW, JACOBSON EL, BENJAMIN RC, MOSS J AND JACOBSON MK. (1989). Quantitative studies of inhibitors of ADP-ribosylation *in vitro* and *in vivo*. *J. Biol. Chem.*, **264**, 4312-4317.

ROBERTS JJ. (1978). The repair of DNA modified by cytotoxic, mutagenic, and carcinogenic chemicals. *Adr. Radiat. Biol.*, **7**, 211-435.

SATOH MS AND LINDAHL T. (1992). Role of poly(ADP-ribose) formation in DNA repair. *Nature*, **356**, 356-358.

SATOH MS, POIRIER GG AND LINDAHL T. (1993). $NAD^+$-dependent repair of damaged DNA by human cell extracts. *J. Biol. Chem.*, **268**, 5480-5487.

SEBOLT-LEOPOLD JS AND SCAVONE SV. (1992). Enhancement of alkylating agent activity *in vitro* by PD 128763, a potent poly(ADP-ribose) synthetase inhibitor. *Int. J. Radiat. Oncol. Biol. Phys.*, **22**, 619-621.

SHALL S. (1984). ADP-ribose in DNA repair: a new component of DNA excision repair. *Adv. Radiat. Biol.*, **11**, 1-69.

SHIBATA D, PEINADO MA, IONOV Y, MALKHOSYAN S AND PERUCHO M. (1994). Genomic instability in repeated sequences is an early somatic event in colorectal tumorigenesis that persists after transformation. *Nature Genet.*, **6**, 273-281.

SKEHAN P, STORENG R, SCUDIERO D, MONKS A, MCMAHON J, VISTICA D, WARREN JT, BOKESCH H, KENNEY S AND BOYD MR. (1990). New colorimetric assay for anti-cancer drug screening. *J. Natl Cancer Inst.*, **82,** 1107-1118.

SMITH MT, EVANS CG, DOANE-SETZER P, CASTRO VM, TAHIR MK AND MANNERVIK B. (1989). Denitrosation of 1,3-bis(2-chloroethyl)-1-nitrosourea by class $\mu$ glutathione transferase and its role in cellular resistance in rat brain tumour cells. *Cancer Res.*, **49,** 2621-2625.

TANO K, SHIOTA S, COLLIER J, FOOTE RS AND MITRA S. (1990). Isolation and structural characterization of a cDNA clone encoding the human DNA repair protein for $O^6$-alkylguanine. *Proc. Natl Acad. Sci. USA*, **87,** 686-690.

TISDALE MJ. (1985). Antitumour imidazotetrazines – XI: effect of 8-carbamoyl-2-methylimidazo[5,1-d]-1,2,3,5-tetrazin-4(3H)-one [CCRG 81045; M and B 39831; NSC 362856] on poly(ADP-ribose) metabolism. *Br. J. Cancer*, **52,** 789-792.

TONG WP, KIRK MC AND LUDLUM DB. (1982*a*). Formation of the cross-link 1-[$N^3$-deoxycytidyl]-2-[$N^1$-deoxyguanosinyl]-ethane, in DNA treated with $N,N^1$-bis(2-chloroethyl)-$N$-nitrosourea (BCNU). *Cancer Res.*, **42,** 3102-3105.

TONG WP, KOHN KW AND LUDLUM DB. (1982*b*). Modifications of DNA by different haloethylnitrosoureas. *Cancer Res.*, **42,** 4460-4464.

WAXMAN DJ. (1990). Glutathione *S*-transferases: role in alkylating agent resistance and possible target for modulation chemotherapy – a review. *Cancer Res.*, **50,** 6449-6454.

WEDGE SR, PORTEOUS JK, MAY BL AND NEWLANDS ES. (1996*a*). Potentiation of temozolomide and BCNU cytotoxicity by $O^6$-benzylguanine: a comparative study *in vitro*. *Br. J. Cancer*, **73,** 482-490.

WEDGE SR AND NEWLANDS ES. (1996*b*). $O^6$-Benzylguanine enhances the sensitivity of a glioma xenograft with low $O^6$-alkylguanine-DNA alkyltransferase activity to temozolomide and BCNU. *Br. J. Cancer*, **73,** 1049-1052.

WEINKAM RJ AND LIN HS. (1979). Reactions of BCNU (1,3-bis(2-chloroethyl)-1-nitrosourea) and CCNU (1-[2-chloroethyl]-3-cyclohexyl-1-nitrosourea) in aqueous solution. *J. Med. Chem.*, **22,** 1193-1198.

WHISH WJD, DAVIES MI AND SHALL S. (1975). Stimulation of poly (ADP-ribose) polymerase activity by the antitumour antibiotic, streptozotocin. *Biochem. Biophys. Res. Commun.*, **65,** 722-730.

YANG J, HSIEH F, LEE P AND TSENG HR. (1994). Strand and sequence-specific attenuation of N-methyl-N′-nitro-N-nitroguanidine-induced G.C to A.T transitions by expression of human $O^6$-methylguanine-DNA methyltransferase in Chinese hamster ovary cells. *Cancer Res.*, **54,** 3857-3863.

YOUNG RC, DEVITA VT, SERPICK AA AND CANELLOS CP. (1971). Treatment of advanced Hodgkin's disease with [1,3-bis(2-chloroethyl)-1-nitrosourea] BCNU. *N. Engl. J. Med.*, **285,** 475-479.

ZELLER WJ AND MAGULL-SELTENREICH A. (1995). Sensitization of human colon tumour cell lines to carmustine by depletion of $O^6$-alkylguanine-DNA alkyltransferase. *J. Cancer Res. Clin. Oncol.*, **121,** 225-229.